# Supplementary material for: Similarities and Differences in the Late-Onset GM2 Gangliosidoses: Tay-Sachs and Sandhoff Diseases
Source: medRxiv. 2025 Aug 8:2025.08.05.25333048. Preprint. [Version 1] doi: 10.1101/2025.08.05.25333048 (PMC12443050; doi:10.1101/2025.08.05.25333048)
Supplement: 1 [file NIHPP2025.08.05.25333048V1-supplement-1.pdf]

medRxiv preprint doi: https://doi.org/10.1101/2025.08.05.25333048; this version posted August 8, 2025. The copyright holder for this preprint (which was not certified by peer review) is the author/funder, who has granted medRxiv a license to display the preprint in perpetuity. This article is a US Government work. It is not subject to copyright under 17 USC 105 and is also made available for use under a CC0 license.

Supplementary Materials

Addendum: The Brief Ataxia Rating Scale (BARS)

Gait

0: Normal
1: Almost normal naturally, but unable to walk with feet in tandem position
2: Walking without support, but clearly abnormal and irregular
3: Walking without support but with considerable staggering; difficulties in half turn
4: Walking without support not possible; uses support of the wall for 10-meter test.
5: Walking possible only with one cane
6: Walking possible only with two canes or with a stroller
7: Walking possible only with one accompanying person
8: Walking impossible with one accompanying person (2-person assist; wheelchair)

Knee-tibia test (decomposition of movement and intention tremor)
(Left and Right scored)

0: Normal
1: Lowering of heel in continuous axis, but movement is decomposed in several phases, without real jerks, or abnormally slow
2: Lowering jerkily in the axis
3: Lowering jerkily with lateral movements
4: Lowering jerkily with extremely long lateral movements, or test impossible

Finger-to-nose test (decomposition and dysmetria of arm and hand)
(Left and Right scored)

0: Normal
1: Oscillating movement of arm and/or hand without decomposition of the movement
2: Segmented movement in 2 phases and / or moderate dysmetria in reaching nose
3: Segmented movement in more than 2 phases and / or considerable dysmetria in reaching nose
4: Dysmetria preventing the patient from reaching nose

Dysarthria

0: Normal
1: Mild impairment of rate/rhythm/clarity
2: Moderate impairment of rate/rhythm/clarity
3: Severely slow and dysarthric speech
4: Speech absent or unintelligible

Oculomotor abnormalities

0: Normal
1: Slightly slowed pursuit, saccadic intrusions, hypo/hypermetric saccade, nystagmus
2: Prominently slowed pursuit, saccadic intrusions, hypo/ hypermetric saccade, nystagmus

TOTAL (out of 30)

Reference: Schmahmann JD, Gardner R, MacMore J, et al. Movement Disorders. 2009: 1820-1828

Supplement Figure 1. The Brief Ataxia Rating Scale (BARS).
